# Role of PhaC Type I and Type II Enzymes during PHA Biosynthesis

**DOI:** 10.3390/polym10080910

**Published:** 2018-08-13

**Authors:** Valeria Mezzolla, Oscar Fernando D’Urso, Palmiro Poltronieri

**Affiliations:** 1 Department of Biological and Environmental Science and Technologies, University of Salento, Ecotekne, 73100 Lecce, Italy; valeria.mezzolla@gmail.com (V.M.); bioesplora@gmail.com (O.F.D.); 2 CNR, Agrofood Department, Institute of Sciences of Food Productions (ISPA-CNR), 73100 Lecce, Italy

**Keywords:** PhaC synthase, classification, dimerization, substrate binding, exit cavity, C3–C14 alkanes, polymer composition

## Abstract

PHA synthases (PhaC) are grouped into four classes based on the kinetics and mechanisms of reaction. The grouping of PhaC enzymes into four classes is dependent on substrate specificity, according to the preference in forming short-chain-length (scl) or medium-chain-length (mcl) polymers: Class I, Class III and Class IV produce scl-PHAs depending on propionate, butyrate, valerate and hexanoate precursors, while Class II PhaC synthesize mcl-PHAs based on the alkane (C6 to C14) precursors. PHA synthases of Class I, in particular PhaC_Cs_ from *Chromobacterium* USM2 and PhaC_Cn_/*Re*PhaC1 from *Cupriavidus necator*/*Ralstonia eutropha*, have been analysed and the crystal structures of the C-domains have been determined. PhaC_Cn_/*Re*PhaC1 was also studied by X-ray absorption fine-structure (XAFS) analysis. Models have been proposed for dimerization, catalysis mechanism, substrate recognition and affinity, product formation, and product egress route. The assays based on amino acid substitution by mutagenesis have been useful to validate the hypothesis on the role of amino acids in catalysis and in accommodation of bulky substrates, and for the synthesis of PHB copolymers and medium-chain-length PHA polymers with optimized chemical properties.

## 1. Introduction

Polyhydroxyalkanoates (PHAs) are biodegradable polyesters produced in several Gram-negative and Gram-positive bacteria, and in archaea and cyanobacteria [1,2,3,4,5,6,7,8]. PHAs are polymers containing various 3-hydroxyalkanaotes, such as 3-hydroxypropionate (3HP), 3-hydroxybutyrate (3HB), 3-hydroxyvalerate (3HV), 3-hydroxyhexanoate (3HHx), 3-hydroxyheptanoate (3HHp), 3-hydroxyoctanoate (3HO), 3-hydroxynonanoate (3HN), 3-hydroxydecanoate (3HD), 3-hydroxydodecanoate (3HDD), in addition to 4-hydroxybutyrate (4HB) or 5-hydroxyvalerate (5HV), and are produced through the availability of the corresponding CoA thioester substrates [9,10,11,12,13,14].

There also exist PHAs with unsaturated monomers (3-hydroxyalkenoates), produced by Pseudomonas sp. possessing Class II PHA polymerizing enzymes. PhaC synthases are grouped into four classes based on substrate specificity, and the preference in forming short-chain-length (scl) or medium-chain-length (mcl) polymers: Class I, Class III and Class IV produce principally scl-PHAs, while Class II PhaC synthesize mcl-PHAs [7,15].

The PHA biosynthesis genes include: *PhaPs*, genes coding for phasins, the granule-assembling proteins; *PhaM*, encoding the activator and accelerator of the catalytic activity of PHA synthase, encoded by *PhaC*; *phaA*, encoding the acetoacetyl-CoA 3-ketothiolase; *phbB,* coding for the acetoacetyl-CoA reductase; *phaG*, coding for 3-hydroxyacyl-carrier protein-CoA transferase; *phaJ*, encoding the enoyl-CoA hydratase; and *PhaZ*, encoding the intracellular PHA-depolymerizing enzymes.

PhaC proteins possess the so-called lipase box, G-X-S-X-G, and a secondary structure containing the α/β hydrolase fold, a characteristic succession of alpha helices and beta strands, typical of lipases. In PhaC enzymes, the lipase box has the conserved sequence G-G/S-X-C-X-G/A-G, renamed the PhaC box consensus sequence. The Cys in the lipase box-like sequence is the catalytic amino acid, forming the covalently bonded intermediate, Cys-S-H3B [7,16].

PHA synthase activity is based on the catalytic triad C-H-D, cysteine, histidine, aspartate, also responsible for catalysis in lipases (S-H-D). In the catalytic triad, the negatively charged Asp_447_ in PhaC_Cs_ from *Chromobacterium* spp. [17,18] and Asp_480_ in PhaC_Cn_ from *Cupriavidus necator* [19,20] assist to enhance the basicity of His_477_ and His_508_, respectively, by formation of a direct hydrogen bond. Previous reports suggest that the Asp residue of the triad acts as a general base catalyst to accelerate deprotonation of the 3-hydroxyl group of HB in the step involving elongation of the PHA product.

PHA synthases are grouped into four classes based on the kinetics and mechanism of reaction. The grouping of PhaC enzymes into each class is dependent on the structure of the PhaC, alone or in association with other subunits, and the substrate specificity: Class I, Class III and Class IV produce scl-polymers depending on 3-hydroxypropionate (3HP), 3-hydroxy- and 4-hydroxybutyrate (3HB, 4HB), 3-hydroxyvalerate (3HV) and 3-hydroxyhexanoate (3HH) precursors (C3 to C6 carbons), while Class II PhaC enzymes synthesize mcl-polymers depending on 3-hydroxyhexanoate (3HH), 3-hydroxyheptanoate (3HHp), 3-hydroxyoctanoate (3HO), 3-hydroxydecanoate (3HD), 3-hydroxyundecanoate (3HUD), 3-hydroxydodecanoate (3HDD) (C6 to C12), and availability of the corresponding CoA thioester substrates, originating from three different metabolic pathways [9,10,20].

While some bacterial species produce mainly PHB homopolymers consisting only of 3HB monomers, other species can synthesize various PHAs, depending on availability of intermediate precursors [1,3,9]. The structure and properties of the polymers are affected by the monomers that are incorporated. PHAs can be molded into films and hollow bodies. Polyhydroxybutyrate (PHB) is brittle, fragile and stiff, with low elongation ability, and a break point below 15%. The incorporation of 3-hydroxyvalerate or other comonomers can decrease the brittleness of PHB. The thermal, rheological and barrier properties of PHAs show good application potential in thermoplastic materials. The synthesis of copolymers is a frequent strategy to improve the properties of PHAs, to improve the plastics flexibility and lower the glass transition temperature (*T*_g_) and the melting temperature (*T*_m_) [21]. P3HB-4HB polymers and conventional thermoplastics used for packaging show high tensile strength and higher elongation at break. mcl-PHAs containing both 3HB and 3HV monomers are elastic, have low melting point, a relatively low degree of crystallinity, and various tensile strengths. PHB copolymer containing 3-hydroxyvalerate unit P(3HB-*co*-3HV) has been developed with improved mechanical properties. To this aim, either optimization of substrate availability (feedstock, successive addition of precursors) or efficiency of enzymes, through genetic engineering and selection of PHA synthases, have been applied.

### 1.1. Class I and Class II PHA Synthases

Classes I and II PHA synthases are formed by a single protein (PhaC), of about 60 kDa.

In Class I, the active PhaC enzyme is a dimer, with catalytic (CAT) domains facing each other, and with N-domains making direct contacts, sustaining protein interaction and dimerization. It was hypothesized that a partially folded catalytic domain is partially occupied by the lid-and-cat domain secondary structure, which changes its conformation in the presence of activation factors to open the catalytic domain and to allocate the 3HB-CoA to its binding site [17].

**Figure 1:**
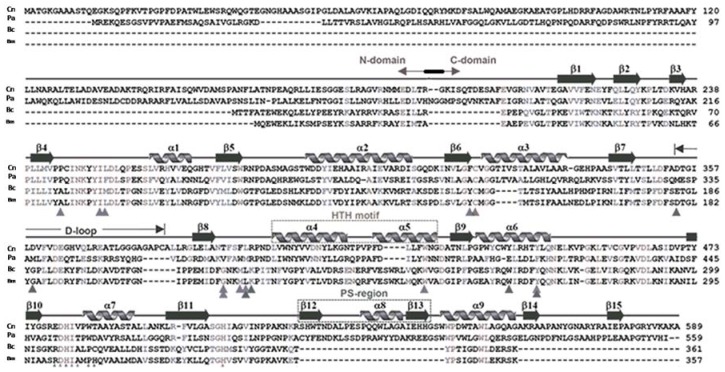
Amino acid sequence and secondary structure of *Cupriavidus necator* PhaC_Cn_, aligned with the PHA synthase of Class II (*P. aeruginosa*), Class IVm and Class IVb, from *Bacillus megaterium* and *B. cereus*, respectively.

PhaC sequences differ in their length. In *Cupriavidus necator* (formerly *Ralstonia eutropha*) PhaC_Cn_/*Re*PhaC, a Class I enzyme, the sequence is composed of a 191 amino acids proteolytically cleaved at the N-terminal domain after arginine, and a C-domain, containing the catalytic site, composed of 398 amino acids, for a total of 589 residues (Figure 1). In *C. necator*, PhaC_Cn_ contains Cys_319_, Asp_480_ and His_508_, located between the beta-6 and alpha-3 turns, the beta-10 and alpha-7 turns, and at the end of the beta-11 strand, respectively. In between Cys_319_ and Asp_480_ is located the D-loop and the helix-turn-helix motif (HTH), formed by α4, α5, α6 and β7–β8 stretches [18,19]. PhaC_Cn_/*Re*PhaC1 was also studied by X-ray absorption fine-structure (XAFS) analysis, which confirmed the previous findings on the protein assembled as a dimer [22].

The PhaC synthase from *Chromobacterium* spp., PhaCPhaC_Cs_, was extensively studied [16,17]. PhaC_Cs_ has a peculiarity of utilization of 3HB, 3HV and 3HH, producing scl-PHA polymers with mixed composition, with the ability to incorporate C5 and C6 alkanes into the PHA polymer. PhaC_Cs_ was found to be highly active, with fast polymerization rate [17]. PhaC_Cs_ is shorter in length (by about 29 amino acids) with respect to PhaC_Cn_ and this produces differences in the numbering of amino acids. The PhaC_Cs_ structure has a substrate-binding site hidden by a partially disordered protein domain, the CAP domain [16,17]. Cysteine_291_, at the end of the ß6-sheet, is followed by the CAP domain, containing the LID structure, which closes the accessibility of the substrate access pocket. The β-sheets in the CAP domain have been renumbered with Greek letters in the PhaC_Cs_ sequence. Thus, from the N-terminal sequence, up to the β6–α3 turn, the two PHA synthases conserve the same numbering in their secondary structures, but the successive α–β turns are differently numbered. In PhaC_Cs_, the core subdomain contains six ß-strands (ß8 to ß13) and four a-helices (α4 to α7), whose numbers do not correspond to those in the PhaC_Cn_-CAT structure.

In PhaC_Cs_, the catalytic triad is composed of Cys_291_, His_477_ (located after the β9-sheet, with respect to the β11-sheet in PhaC_Cn_) and Asp_447_ (located at the turn formed by the ß8-strand and α4-helix, the ß8–α4 fold, in respect to the β10–α7 fold in PhaC_Cn_).

In other species, such as *Delftia acidovorans* (previously *Comamonas acidovorans*), the PHA synthase contains a large insert of 40 amino acid residues shown to improve the specific activity of the enzyme, located in the α/β-hydrolase fold, following the catalytic cysteine after the β6 turn [23].

*Aeromonas* spp., such as *A. caviae*, *A. hydrophila* and *A. punctata*, possess PhaC enzymes belonging to Class I. These organisms have synthases of type I with mixed substrate range, which enable the production of “Nodex” mixed-type scl-mcl-PHAs by these bacteria [24].

The enzyme PhaC_Cc_ from *Caulobacter crescentus* (*C. vibrioides*) [25] was displayed to accommodate 3-hydroxyalkanoates with various alkyl side-chain lengths.

Class II PhaC synthases have been extensively studied, and are widely distributed in bacteria: in *Pseudomonas* spp. (*P. putida*, *P. mendocina*, *P. oleovorans*, *P. campisalis*, *P. stutzeri),* there are two PhaCPhaC genes, of which PhaC1 is the active enzyme under physiological conditions. PhaC synthases have been reported in *Halomonas* spp., such as *H. campisalis*, *Halomonas* sp. O-1 and *Halomonas elongata* DSM2581 [26], and in *P. stutzeri*, and they can be exploited in polymerization of mcl-PHAs [27]. There are two PHA synthases, PhaC1 and PhaC2, in *P*. *oleovorans*, of which PhaC2 has a higher affinity for 3-hydroxyhexanoate (3HH) monomers.

Class II PhaCPhaC enzymes differ from PhaC_Cn_, as prototype of Class I PHA synthases, by about 28 amino acids, reaching the C-terminal (1–559) with a sequence shorter by about 30 amino acids. The catalytic triad has been renumbered as Cys_296_, Asp_452_, His_453_ and His_480_ in *Pseudomonas* spp., prototype for Class II PHA synthases.

### 1.2. Class III and Class IV PHA Synthases

Class III PHA synthases are made of two subunits, namely, a catalytic subunit PhaC (40–53 kDa) and a second subunit PhaE (ranging from 20 to 40 kDa), which form the PhaEC complex, in which the PhaE subunit is necessary for PHA polymerization. Class III PhaC are structured as tetramers, such as phaEC from *Chromatium vinosum* (with catalytic triad Cys_149_, Asp_302_, His_331_), whose enzyme activity has been studied using substrate analogs [15]. The authors performed molecular docking and in-silico studies that are in agreement with the crystal structure of synthases available [15], based on homology models built using CPHmodels3.0, SWISS-MODEL and I-TASSER, performing structure-guided sequence profiles. The results of their analysis, referring to PhaC_Cn_ and PhaC Class III from *Chromatium vinosum*, describe the presence of an active site of cysteine that is buried in a pocket. The authors, by comparison with other enzymes with known crystal structures (lipases), postulated the presence of the substrate-entrance and product-exit channels [15].

An interesting enzyme representative of Class III has been isolated and characterized in *Thiocapsa pfennigii* [28]. There is also an archaeal type, PhaC Class IIIA [29]: this group is represented by *Haloarcula marismortui*. Archaea present good perspectives of exploitation for polymer production, given by the easiness of PHB extraction.

Class IV PHA synthases from *Bacillus* spp. are composed of a catalytic subunit PhaC (41.5 kDa) and a PhaR (22 kDa) subunit, similarly to Class III synthases composed of PhaE and PhaC units. Class IV PHA synthases are classified as *Bacillus cereus* type (IV_c_) [30], B. megaterium type (IVm) [31], and *B. bataviensis* type (IV_b_), with 33% homology to the other PhaC sequences [5]. In *E. coli* expressing PhaRC from *B. cereus* YB-4, the biosynthesized PHA undergoes synthase-catalyzed alcoholytic cleavage using endogenous and exogenous alcohols. This alcoholysis is thought to be shared among Class IV synthases, and this reaction is useful for regulation of PHA molecular weight and for modification of the PHA carboxy terminus.

The catalytic cysteine in the active site is C_151_ in *B. cereus*, and C_147_ in *B. megaterium* type IV enzymes. As shown for PhaCYB4 from *B. cereus* YB-4, the involvement of Cys_151_, Asp_306_ and His_335_ in polymerization activity was shown by site-directed mutagenesis [5].

### 1.3. Diversity and Spread of PhaC in Bacteria

PHA synthase genes can be identified in environmental bacterial strains for a preliminary screening, before knowledge of PHA synthesis ability due to the presence of the gene, using PCR amplification with conserved primers [7,32]. Through PCR analyses, *PhaC* genes were detected in a collection of bacterial strains isolated from soils and from marine environments. In samples of environmental strains, we amplified *PhaC* gene sequences in colonies from environmental isolates, and performed DNA sequencing of ribosomal DNA to identify the strains at species level: with this method, several species were classified for their ability to produce PHA, for instance, *P. oleovorans*, *P. fluorescens*, *P. sihuiensis*, *P. putida*, *Comamonas testosteroni*, *Aeromonas hydrophila*, as well as *Cupriavidus necator*. It is envisaged that PCR screening using various primer sets can be optimized to find new PhaC polymorphisms and potential novel PHA synthase sequences. Quelas reported the presence in *Bradyrhizobium japonicum* USDA110 of five polyhydroxyalkanoate (PHA) synthases (PhaC), distributed into four different PhaC classes [33], and characterized the requirements for two of the genes in legume nodules under various physiological conditions.

## 2. Crystal Structure

In two publications that appeared almost simultaneously, two teams reported on the crystal structure of PhaC_Cn_-CAT, the catalytic domain of PhaC from *Cupriavidus necator* [18,19,24]. PhaC_Cn_-CAT was shown to dimerize, and to adopt a partially open form maintaining a narrow substrate access to the active site. PhaC_Cn_ needs PhaM, the primer of PHA synthesis, to start and accelerate polymer synthesis, and this may be due to increased accessibility of 3HB-CoA substrate to the active site. Wittenborn obtained the crystal structure of PhaC_Cn_(C_319_A), a construct in which the active site cysteine (Cys_319_) was mutated to alanine to improve protein stability in the absence of detergent. During the crystallization, proteolysis of PhaC_Cn_ occurred after the N-domain (R_192_), leading to the crystal structure of the C-domain: PhaC_Cn_-CAT is formed by two core subdomains (G_143_–F_352_, L_450_–A_589_), flanking on both sides a dimerization domain (A_353_–L_549_) containing the dimerization loop (D-loop) and the helix-loop-helix (HTH) domain; in addition, in the terminal core subdomain there is an extended C-terminal region (EC: R_521_–A_589_), which is missing in Class IV PhaC (Figure 1), and a protruding structure, PS, that elongates from the extended C-region.

The catalytic domain of PhaC_Cn_ contains the residues 201–368 and 378–589 (with residues 369–377 devoid of any structure), showing an α/β-hydrolase fold, featuring a central mixed β-sheet flanked by α-helices on both sides. This architecture is similar to that seen in lipases. The CAT domain in the PhaC_Cn_ sequence is structured by the presence of the β1–4-sheets, the α1-helix, the β5-sheet, α2-helix and β6-sheet facing the lipase box, followed by the α3–β7 fold: after this structure there is the D-loop; after the β8-sheet, there is the helix-loop-helix (HTH), composed of the α4- and α5-helices facing each other, and the β9–α6 fold, where the dimerization subdomain ends (L_449_); as for the other amino acids of the catalytic triad, the aspartate is located between the β10–α7 fold, and the histidine is located after the β11-sheet. The active site of PhaC_Cn_ is accessible via a water-filled channel, with a size of 12.5 Angstroms, which can accommodate the 3HB-CoA substrate and/or short PHA oligomers.

Two PhaC monomers interact through the dimerization surfaces (A_353_–E_445_), containing hydrophobic amino acids, by means of interaction between one monomer helix-loop-helix motif (HTH) and the D-loop of the second monomer [18,19].

In the report on the crystal structure obtained from the catalytic domain of PhaC from *Chromobacterium* sp. USM2, PhaC_Cs_-CAT was compared to the PhaC_Cn_-CAT crystal structure [17]. Considering the two structures described, in PhaC_Cs_-CAT, a difference in the accessibility of the active site has been evidenced. Chek showed that in the PhaC_Cs_-CAT dimer, the CAP and LID domains close the access to the substrate binding site [17]. The structure proposed by Chek and colleagues describing a PhaC_Cs_ active site covered by the CAP subdomain differs from the partially open form of the PhaC_Cn_ catalytic domain reported by Wittenborn. The CAP domain occupies partially the access to the substrate binding pocket, and the LID domain needs to slide away in order to free the access for 3OH-alkanoyl-S-CoA units. Both catalytic domains of PhaC_Cs_ and PhaC_Cn_ form a dimer mediated by the CAP subdomain. The difference between the closed and partially open forms is provided by the conformation of the CAP subdomain. The CAP subdomain undergoes a conformational change during catalytic activity with rearrangement of the dimeric form.

The main difference between the two crystal structures was found in the folding of αB’- and ηB’-helices and their linker loop of PhaC_Cs_-CAT, while the corresponding positions in PhaC_Cn_-CAT show a long α4-helix that presents a partial access to the active site. The region Leu_402_–Asn_415_ forming the α4-helix in PhaC_Cn_-CAT is conserved among Class I and II PHA synthases, whereas the corresponding segment, Leu_369_–Lys_382_ of PhaC_Cs_-CAT, displays a disordered structure.

## 3. Catalytic Mechanism

The models proposed for the available PhaC structures hypothesize the presence of a substrate entrance tunnel that accommodates HB-CoA, with a size of about 12.5–13 Å, and a product egress tunnel, positioned perpendicularly to the entrance tunnel. Various catalytic mechanisms for PHA synthases have been proposed, in the context of dimerization of PHA synthases of Class I and II [17].

One mechanism is referred to as the nonprocessive ping-pong model: this mechanism requires two cysteines in the active sites in the dimer, for PHA chain elongation, with chain transfer from one cysteine to the second active site. The ping-pong mechanism requires two thiol groups located at a distance short enough to shuttle back and forth the growing (3HB)*n* chain between the two thiols. The dimeric structures described by Wittenborn and by Kim for PhaC_Cn_-CAT, and by Chek for PhaC_Cs_, show that the two active sites are too distant (33 and 28.1 Angstroms, respectively) for successive chemical reactions.

The distance between the active sites in the dimer seems to favor the mechanism based on a single active site for each elongation reaction. In the model described by Chek, the dimer, composed by two units of PhaC*_Cs_* through the contacts between the two CAP domains and the two N-domains, presents two channels leading to the two active sites. The dimeric structure proposed by Chek [17] favors the involvement of one active site for each processing step. In the model, the substrate enters the substrate-binding tunnel, while the chain product is elongated along a path near the protein surface, with a sliding mechanism of the PHA polymer under synthesis along a V-shaped cavity within the enzyme. In the proposed structure, the enzyme moves along the extremity of the forming polymer to add new 3HB units, rather than hosting the polymer into a product egress channel. The mechanism involves a processive model that requires a single active site for PHA chain elongation and a noncovalent intermediate, in addition to a covalent intermediate bound to the Cys residue at the active center during the catalytic cycle. The enzyme dimer, through interactions with other partners, substrates, phasins and phaM, moves the CAP domains to flip away, opening the active site entrance, and freeing the product channel, and the two core units simultaneously accept the substrate and produce the 3HBn polymers. The process occurs with a two-step catalysis mechanism that allows the intermediates to be located in the enlarged cavities partially freed from the CAP occupancy. The arrangement of the dimer, different from that of the PhaC*Cn*-CAT dimer, may allow the CAP subdomains to undergo a conformational change during catalytic activity with rearrangements in the dimer that facilitates substrate entry, intermediate product formation, and product exit from the active site. According to the crystal structure of the PhaC_Cn_-CAT dimer [18,19], the substrates enter through the substrate-binding tunnel: the first 3HB-CoA is attacked by the nucleophilic Cys-SH to produce a 3HB-Cys covalent bond, as in the aforementioned model, and frees CoA-SH, which is released from the product egress tunnel. A second 3HB-CoA attacks 3HB-Cys thioester bond with the hydroxyl group in 3HB to produce a (3HB)2-CoA intermediate, a reaction that frees the Cys residue in the active center. The Cys residue again attacks the thioester bond of the (3HB)2-CoA intermediate to produce (3HB)_n__+__1_, covalently bound to the Cys residue, and releases free CoA. In this model, the growing 3HB polymer is bound to the enzyme at the end of each cycle. This model cannot allow it to position large molecules such as the (3HB)n-CoA intermediate within the substrate binding site, which has a cavity of 12.5 Angstroms.

An alternative model has been proposed with a succession of slightly different reactions. The model proposed for PhaC_Cn_, by Wittenborn, implies that newly entered 3HB-CoA produces 3HB-Cys; then, (3HB)_2_-CoA enters the active site to produce (3HB)_3_-CoA, which is again released from the active site. When a new (3HB)_2_-CoA substrate binds, the 3HB 3-hydroxyl group is deprotonated by His_508_, facilitated through modulation of the histidine basicity by Asp_480_. The newly formed HB alkoxide attacks the Cys-HB thioester, generating a noncovalent, CoA-bound intermediate. However, if the (3HB)_3_-CoA produced is held in the active site and attacked by the active Cys residue again to produce (3HB)_3_-Cys, chain elongation would then require an intersubunit reaction. Again, (3HB)_n_-Cys adducts would require a larger active site cavity.

## 4. Mutation and Amino Acid Substitution Studies

Several studies focused on PHB synthases with mutations enabling the enzymes to accelerate the reaction kinetics [34,35,36,37] and ability to accept bulk substrates as precursors for the production of mcl-PHAs and grafted copolymers.

Nomura and Taguchi [37] reviewed the attempts to engineer various classes of PHA synthases, either by mutagenesis or by evolution, in Class I and Class II enzymes. The methods utilized either random mutagenesis, intragenic suppression mutagenesis, gene shuffling, random mutagenesis combined with site-specific saturation mutagenesis and recombination, localized semirandom mutagenesis, PCR-mediated random chimeragenesis, intragenic suppression mutagenesis, or site-specific saturation mutagenesis.

Many authors described mutations in amino acids positioned in various domains of different PHA synthases, most often finding a decrease in production of mcl-PHA and higher synthesis of scl-PHA. Beneficial effects of mutagenesis studies of Glu_130_ and Ser_477_ have been described [38,39,40,41]. For instance, the E_130_D substitution and S_477_X mutation in type II PHA synthase showed an enhancement of PHA production and alteration of polymer molecular weight.

A mutagenesis study of Class I PHA synthases showed that the F_420_S mutation in PhaC_Cn_ increased the specific activity with a shortened lag phase [42]. This residue corresponds to Phe_387_ of PhaC_Cs_, which is conserved among Class I and II PHA synthases, and is located in the αC-helix of the CAP domain. Phe_387_ is involved in dimerization by participating in an intermolecular nonpolar interaction linking the αC-helix to the LID region, suggesting that the mutation may affect the conformational stability and/or conformation transition of the LID region.

The CAP subdomain provides αC- and αD-helices as building blocks of the active site cavity filled with a cluster of water molecules. In the structure obtained by Chek [17], the C-terminal portion of the LID region of the CAP subdomain is disordered and is followed by αC-helix docked to the core subdomain. Two highly conserved residues, Trp_392_ and Asp_395_, are present in the αC-helix. Trp_392_ of PhaC*Cs* is located in the αC-helix of the CAP subdomain and faces site B of the channel.

A mutational study of PhaC*_Cs_* reported by Chuah [43] showed that in PhaC*_Cs_*, Ala_479_ is a critical residue required for substrate specificity, as determined by various site-specific mutational assays both in vivo and in vitro, and production tests of copolymers such as P(3HB-*co*-3HHx). In PhaC_Cn_ and in other Class I enzymes, this position corresponds to the conserved residue Ala_517_. In the structure proposed by Chek, Ala_479_ is located within the α5-helix, and the side chain protrudes into a depression of the molecular surface formed by loops (β4–α1, β9–α5 and α5–β10 loops) from the core subdomain, and is partially covered by the helix ηB’ and the following loop of the LID region from the CAP subdomain. The A_479_ mutation results in weakening of the interactions between the LID region and the core subdomain, and stabilizes the active form of this enzyme by releasing the LID region from the active site. Since Ala_479_ is surrounded by polar residues (Ser_475_ and Arg_490_), it is supposed that replacement of Ala_479_ with Ser or Thr facilitates hydrogen-bonding interactions with the polar residues and stabilization of the α5-helix harboring the active residue His_477_, important for enzyme activity.

Amara and Rhem attempted to modify the activity of PhaC from *Pseudomonas* species [40]. The conserved residue Trp_398_ was replaced, for example, to Trp_398_Phe and Trp_398_Ala, and the mutation resulted in inactivation of the enzyme. Using the threading model of enzyme structure, the authors located the Trp residue as exposed on the surface, in agreement with the results shown by Chek for Class I enzymes [44,45,46,47,48,49].

Tyr_412_ in PhaC_Cs_, and Tyr_446_ in the α6-helix in PhaC_Cn_, are residues conserved in Class I, III and IV PHA synthases, while Phe occupies this position in Class II synthases; in addition to this amino acid position, there is a second substitution that seems to have a role in accommodating larger substrates. Tyr_438_ is conserved in Class I, III and IV enzymes, while in Class II PhaC this position is occupied by His: this may contribute to a reduction of size, eliminating the bulky side chain (phenol ring), and determining changes in polar interactions with other amino acids facing the substrate entrance tunnel; these amino acids’ interactions may account for the property to accommodate large substrates in Class II enzymes.

PhaC1 and PhaC2 from *Pseudomonas stutzeri* [42] have been applied to produce mcl-PHAs in engineered bacteria. *Ps*PhaC2 with four point mutations, at E_130_D, S_325_T, S_477_G and Q_481_K, was used to accommodate substrates with various shapes and structures, to produce mcl-PHAs and block copolymers. The putative catalytic residues Cys_296_, Asp_452_, His_453_ and His_480_ were replaced by site-specific mutagenesis [38,39]. Considering the *Pseudomonas* mcl-PHA synthases, the His_480_Gln substitution did not affect enzyme activity, posing the doubt that His is not a component of the catalytic triad. As for a second conserved histidine, when His_453_ was replaced by Gln, the modified enzyme showed only 24% of wild-type in-vivo activity, which makes one suppose that His_453_ might be part of the catalytic triad in Class II PHA synthases [40]. However, no other study confirmed the involvement of His_453_ in Class II PhaC2 catalysis.

Sheu studied the increase of PHA synthase thermostability and activity, using chimeric constructs, indicating that some amino acid substitutions may stabilize the enzyme at higher temperature [34].

## 5. Production of PHA in Fermentors

Various companies are involved in production of bioplastics for industrial applications. The methods are various, either using patented strains, engineered PHA synthases, or growth conditions favoring the high yield and high PHA content/dry cell weight. Recently, a fine review has been published on this topic [50]. In the field of monitoring the endpoint step of PHA synthesis, and bacteria collection, various methods have been established, from lipid staining [51] and analysis of fluorescence intensity, to physic-chemical analyses (Raman, FTIR spectra). Since bacterial cultures require sterilization that is costly at industrial scale, methods based on halophilic strains have been proposed to circumvent the sterilization process. Extraction of PHAs from bacteria requires costly procedures, and therefore, researchers used archaea or cyanobacteria that have PHA granules easily extracted, decreasing the costs of production [52,53].

## 6. Progress and Advancements in the PHA Field

Recent advancements in PHA granule structure and composition have been achieved [54].

PHA granules harbour a considerable number of proteins on their polymer surface, suggesting that they represent supramolecular complexes with specific functions. The high-molecular-weight storage PHB consists of >10^3^ 3HB residues (storage PHB). PHB granules in vivo are covered by a surface layer that is distinct from the polymer core. PHA granules are structured through the action of various proteins on the surface. As an analogy to organelles, the functional granules were proposed to be named carbonosomes by Jendrossek and Pfeiffe, although this term has not been widely accepted. PHA granules represent supramolecular complexes with specific functions. In addition to phasins (such as PhaP2, PhaP3, PhaP4), among the proteins identified during PHA granule isolation, there are the PHB synthase (PhaC1), PhaM, the activator of PhaC, acetyl-CoA acetyltransferase, and acyl-CoA synthetase: their presence may be explained by the need to avoid accumulation of CoA-SH, produced by the PHA synthase during polymer synthesis, since an excess of CoA would inhibit the enzyme. The most accurate model for PHA synthesis within bacterial cells is the Scaffold Model: it assumes that PHB synthase of nascent PHB granules is attached to a scaffold within the cell. PHB granules have been localized in the cell centre, along with the length axis of the bacteria. PhaM, which specifically interacts with PhaC1 and with phasin PhaP5, interacts also with DNA and with the nucleoid in vitro and in vivo, and this may explain why PHB granules have been found attached to the bacterial nucleoid.

## 7. Conclusions

In this review, we reported on the classification of PHA synthases, the proposed structures and roles of individual amino acids in the catalysis, and mechanism of activity of Class I and Class II PHA synthases, presenting the information available on the other types of enzymes. We have reviewed the engineering attempts and the effect of modification of key amino acids on the enzymatic activity and product formation. It is expected that PHA synthases may be further improved to produce effectively and at convenient costs tailor-made polymers.
